# African Primary Care Research: Participatory action research

**DOI:** 10.4102/phcfm.v6i1.585

**Published:** 2014-03-05

**Authors:** Bob Mash

**Affiliations:** 1 Family Medicine and Primary Care, Stellenbosch University, South Africa

## Abstract

This article is part of the series on African primary care research and focuses on participatory action research. The article gives an overview of the emancipatory-critical research paradigm, the key characteristics and different types of participatory action research. Following this it describes in detail the methodological issues involved in professional participatory action research and running a cooperative inquiry group. The article is intended to help students with writing their research proposal.

## Abstrait

**La recherche sur les soins de santé primaire en Afrique: la recherche sur l'action participative**.

Cet article fait partie de la série consacrée à la recherche sur les soins de santé primaire en Afrique et traite essentiellement de la recherche sur l'action participative. L'article donne un aperçu du paradigme de recherche émancipatoire-critique, les caractéristiques fondamentales et les différents types d’études sur l'action participative. Elle poursuit en décrivant dans le détail les problèmes méthodologiques qu'implique la recherche professionnelle sur l'action participative et l'organisation de groupes de discussion de manière coopérative. Cet article est destiné à aider les étudiants à rédiger leur proposition de recherche.

## Introduction

Participatory action research (PAR) is particularly suited to the primary care context, not only because of its tradition of working with communities, but also because of its ability to improve clinical practice.^1, 2^ Primary care services, by their nature, are close to the communities that they serve and questions often arise regarding how to work with community groups in order to address the underlying determinants of ill health. Likewise, questions often arise about how to improve clinical practice or the performance and organisation of the health system. Participatory action research is well suited to bridging the gap between evidence and practice, where evidence produced in clinical trials or expressed in national guidelines must be implemented in and adapted to a particular context. This article will describe the essential characteristics of PAR with the intention of helping the reader to develop a research proposal using this study design. The general approach to writing a research proposal has been described in the first article in the series and the structure used in that article is adopted here.^3^

## The emancipatory-critical paradigm

Before getting to the more practical details of study design it is important to recognise that PAR requires the reader to embrace a different research paradigm, with different values and assumptions to the more orthodox empirical-analytical paradigm of most medical research.^4^ PAR as a methodology is embedded within this emancipatory-critical paradigm (ECP). Table 1 compares the essential characteristics of these different research paradigms. At heart, the ECP is about creating new knowledge by transforming or changing the world in which the research is embedded and reflecting critically on what is learnt in the process. People in the ECP are neither objects to be measured, nor subjects to be understood, but are rather participants in both action and research. Contrary to conventional research, the researcher himself is also a participant in and not an observer of the research process. New knowledge in the ECP is generated as a consensus of the participant's learning and participants may use both quantitative and qualitative techniques in this process. In the ECP, research usually starts with a question regarding how a particular problem can be solved and the participants will align themselves with solving this problem. In doing this, they may generate additional research questions and in some cases even redefine the nature of the problem as the process unfolds. The ECP closes the gap between evidence and practice as learning is immediately put into practice as part of the process. The knowledge that is generated by the research process is highly contextualised and cannot be generalised automatically to other contexts. The concept of transferability applies in that readers of research articles in this paradigm must decide on which findings can be transferred to their own context. The complexity of the specific community or health system in which the research is embedded makes it difficult to assume that the application of what has been learnt will work automatically in another context.

**TABLE 1:** The emancipatory-critical paradigm.

Paradigm	Empirical-analytical	Interpretative-hermeneutic	Emancipatory-critical
Relationship of researcher to ‘reality’	Testing and measuring	Exploring and interpreting	Changing and transforming
View of the researched person	Object to be measured	Subject to be understood	Participant in the process
View of truth	Correspondence to the facts	Coherence within the data	Consensus of each person's learning
Research process	Predominantly quantitative measurements	Predominantly qualitative measurements	Participatory using both quantitative and qualitative techniques
Research question	Fixed hypothesis Set by the researcher	Open-ended question Set by the researcher	Open-ended question Negotiated with group and can evolve
Implementation of results	Recommendations made for action by other people Generalisable	Insights offered for use by other people Transferable	Findings implemented as part of the research Transferable

For medical practitioners to embrace this paradigm fully it usually requires them to undergo a journey in which they unlearn many of the positivist values and assumptions and become comfortable with trusting the participatory process. Many of these lessons are learnt by being willing to attempt research with the help of a competent mentor.

## Key characteristics of participatory action research

**FIGURE 1:**
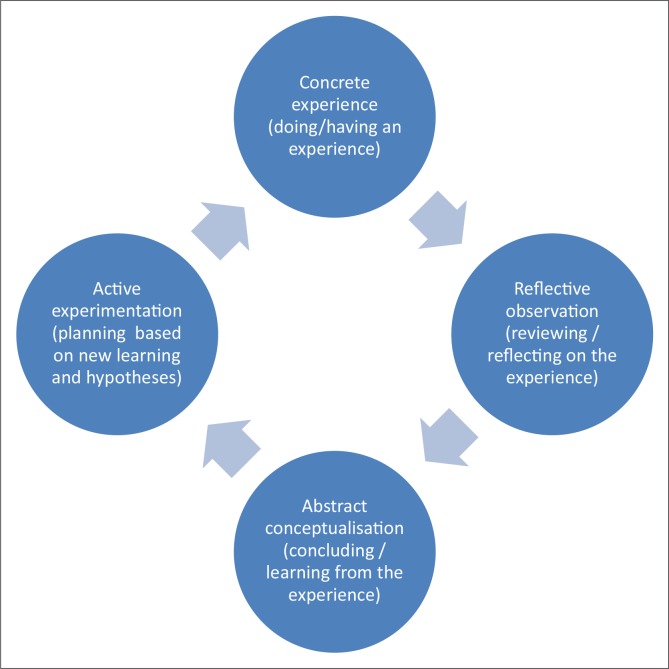
Action-reflection learning cycle. *Source:* Kolb (1984)^8^

Participatory action research can be considered as one methodological approach within this emancipatory-critical paradigm. As the name implies, PAR is an iterative process between action and research.^5, 6, 7^ On the one hand, action requires one to put one's learning into practice, whilst research requires one to reflect and clarify what one has learnt from this experience and to develop new theory and propositional knowledge that is then incorporated into new action. It is participatory and always involves working *with* rather than *on* people. This requires attention to issues of power and hierarchy so that there is a genuinely respectful, open and democratic group process. At its core, PAR believes that people can change their reality and create new knowledge through engaging both consciously and systematically with the steps of the learning cycle as shown in Figure 1.^8^ In this cycle, the participants engage with a process of observing and reflecting on their own concrete experience, agreeing on what has been learnt in the form of new propositional knowledge or abstract concepts and then planning to experiment actively with this new knowledge in another cycle of action and reflection.

Although most academic institutions and scientific journals are most interested in the propositional knowledge that is generated by PAR, it is clear that other types of knowledge will also be created. For example, by changing their practice participants will develop new skills and competencies, which can be seen as practical knowledge and will inevitably also engage with personal growth and change, which can be seen as experiential knowledge.

## Types of participatory action research

PAR is not a homogenous field and there are different traditions within it. Three broad traditions have been identified.^5^

Empowering PAR works with communities to liberate, emancipate and empower them. Proponents of empowering PAR would include people such as Paulo Freire who spoke of the need to develop critical consciousness amongst oppressed communities and to assist them with transforming their reality.^9^ His iconic book, ‘Pedagogy of the Oppressed’,^9^ speaks to this perspective and has also had an influence on the development of adult learning theory. In the context of African primary care research, it is easy to see how empowering PAR may have application within community-oriented primary care and in engaging communities in solving their own health problems.^1^ One example of empowering PAR in the South African context is a group of community members and health workers who looked at how to prevent mortality and morbidity from traditional circumcision in the Eastern Cape.^10^

Organisational PAR has developed in the context of business, industry and the corporate sector so as to solve organisational problems in a more participatory way.

Professional PAR works with professionals who want to change their practice and has been embraced by both the educational and health sectors. Examples of this within the health sector include a group of general practitioners adapting a World Health Organization programme on mental disorders to their South African context,^11^ the leadership of a community health centre trying to develop continuity of care within practice teams,^12^ a group of nurse counsellors adapting a protocol on the recognition and management of intimate partner violence to the South African primary care setting,^13^ or chronic care teams within community health centres trying to improve the annual review of people with diabetes.^14^

The main principles of professional PAR have been summarised in the CRASP model:^15^

Critical collaborative inquiry by

Reflective practitioners being

Accountable and making the results of their inquiry public

Self-evaluating their practice and engaged in

Participative problem solving and continuing professional development

## The cooperative inquiry group

The rest of this article will use the example of a cooperative inquiry group (CIG) to illustrate the key issues that should be covered when writing a PAR proposal.^16^ The CIG is one specific method that has been used to conduct professional PAR in the healthcare sector.^11, 12, 13, 14^

### Research question, aim and objectives

One should define the problem to be solved or the broad question that the group will address. This is often a ‘How to …’ question, for example, ‘How to improve the annual review of people with diabetes in community health centres?’ The research question often arises out of an awareness of the problems encountered in clinical practice – for example, why so many patients with diabetes are controlled poorly.

### Study design

The key characteristics of the research paradigm and PAR study design have been described above and illustrated in Figure 1. In the proposal, after stating the type of study design (e.g. a CIG), the steps of the action-research cycle can be described and illustrated in a figure.

### Selection of participants

A cooperative inquiry group, as with any small group, functions best with 10–15 people. The research proposal must describe the criteria to be used to invite people to join the group and how this process will be facilitated. Sometimes the initiating researcher will make a presentation to the people that might be interested and invite them to come forward, whilst key people may also be invited specifically. The facilitator of the group is usually the initiating researcher who will also function as a group member and participate in the action-reflection process. Members of the group must be able to engage with relevant action and be interested in the topic of the inquiry. In selecting group members, attention should be given to issues of power and hierarchy within the group and how these will be handled, for example, will doctors, nurses and reception staff be able to engage each other in a collaborative and equitable manner. If the group is too heterogeneous it may struggle to function effectively and yet the facilitator can anticipate these issues and plan ways to overcome them. For example, the venue chosen for the group, the names that people use to refer to each other and the way in which contributions are elicited can all help to establish a spirit of respect, equality and openness. Participants will need to give informed consent.

### Cooperative inquiry group process (the intervention)

At the start of the CIG, the researcher must describe how they will go about forming the group and explain the PAR process. Specific training may be needed in order to develop reflectivity in group members. It will be necessary to reach agreement on the research question to be addressed and to be open to modify the question with the group. The group will need to agree on how they will start the inquiry, which often involves developing initial plans for changing practice based on their prior experience and additional information.

In PAR it is not known beforehand what actions the group will engage with or what they will learn. Only the process to be followed can be described in detail. The fundamental process is a cyclical one with continuous cycles of action, observation, reflection and planning in an ongoing spiral format (see also Figure 1). These group members go through these steps in a structured manner:

- *Action*: Putting into action the plans that have been generated from reflection on one's previous experience and understanding of the context.
- *Observation*: Being aware of one's experience and documenting what happened or what has changed; this may include awareness of one's thoughts, feelings and reactions.
- *Reflection*: Reflecting in a structured way on one's observed experience and conceptualising in more abstract forms what one has learnt.
- *Planning*: Incorporating what one has learnt into well-formulated plans for new action at the start of the next cycle.

It should be possible to decide how long the research will last and how many such cycles you anticipate having. All of the steps can be completed as an individual, but reflection and planning should involve the group meeting together to share observations and reflect and plan collectively.

### Documentation of the group process (data collection)

It is important to describe how you will document each part of the process. At an individual level people should keep a personal journal in which they regularly record what they have done, what happened and their thoughts, feelings and reactions. People may also reflect at an individual level and record new concepts or ideas to share with the group.

When the group meets at the beginning and end of each cycle, the group's feedback, reflections and planning should be recorded. This can be done using an audio tape, newsprint and/or field notes. It is often helpful for the facilitator to write a summary of the meeting and circulate this soon afterwards.

Sometimes the group's actions generate their own data, for example, if the group decides to perform interviews or administer a questionnaire. Data will need to be analysed and presented for reflection in the group meeting. The researcher should ensure that the data are captured and stored and the results are documented.

### Building a consensus of the group's learning (data analysis)

At the end of the inquiry process, the group should build a consensus of what they have learnt in a participatory process. This may involve looking back over the whole inquiry process and the various summary documents. Each person should contribute in terms of what they have learnt. Such a consensus does not imply that the group should just summarise everything that they agree on. The consensus can include learning that appears seemingly contradictory. This is possible because it may not yet be apparent how these ideas fit together to make sense of their experience and the group should not reject aspects of their learning for this reason. Consensus can be built through group discussion, but sometimes more formal techniques such as the nominal group technique can be used to ensure a fair and democratic final process. How you plan to facilitate this process should be described.

In the event that the researcher must also write up the CIG for their thesis it is not unusual for the researcher to perform a further analysis on all the qualitative and quantitative data collected once the group has finished meeting. However, their final interpretation of the group's findings should be checked explicitly with the whole group so as to ensure that they remain a valid representation of the group's learning.

### Quality in cooperative inquiry

Orthodox approaches to the appraisal of quality cannot be used in cooperative inquiry because concepts such as bias, confounding and chance do not apply. Concepts used in qualitative research such as triangulation, respondent validation, thick description, reflexivity and deviant case analysis may not be sufficient, although some of them can be relevant to the CIG. For example, it is possible to triangulate the findings from the CIG with evidence from other sources, such as the results of the audit of clinical practice confirming that change has taken place. The following criteria have been proposed for the appraisal of cooperative inquiry:^16^

- *Alignment with purpose*: Alignment of the group members with the purpose of the research both drives the process and acts as the contract between the members. Aligning oneself with a particular outcome or personal intention, rather than the purpose of the research, may lead to a lack of openness in the inquiry.
- *Ownership of the inquiry process*: Ownership of the research by members of the group is crucial to the quality of the inquiry. The initiating researcher will need to transfer power, knowledge of the research methodology, ownership of the research questions and process so that after the group is established he or she does not dominate the inquiry.
- *Development of reflectivity*: As the members of the group are both the researchers and the researched, the quality of the inquiry will depend on their ability to witness themselves. This requires a reflective stance that is characterised by heightened awareness, open-mindedness, critical questioning and commitment to dialogue.
- *Democratic and collaborative group dynamics and facilitation*: The facilitator must strive for a genuine collaborative and democratic group process. The level of trust will be related to telling the truth without judgement and staying within the common purpose. Breaking this contract with each other leads to a loss of trust and commitment and the facilitator must guard against this.
- *Commitment to practical action and experience*: The group must be committed to a balance of both action and reflection. Some groups may find it easy to take action, but difficult to pause for adequate documentation and reflection. Others may be good at planning and reflecting, but short on actually engaging with the practical action.
- *Documentation of the process*: The following three aspects must be documented: (1) the individual experience and action, (2) the group process and dynamics and (3) the developing reflections, learning and final consensus. When writing an account of the CIG it will be necessary to give a ‘thick description’ of the research process and the way in which the final consensus was reached. How these different aspects will be documented should be described in the research proposal. In terms of publications, the scientific journals are more interested in the final consensus (the findings of the study), but the description of the process will be needed to establish its quality and trustworthiness. Making the results available to the public and being accountable as professionals is emphasised in the CRASP model of professional PAR.
- *Transferability*: Transferability is another aspect of quality whereby the group's findings and context should be sufficiently clearly described to enable readers to understand what aspects of the inquiry can be appropriated to their own context. The reporting of this research should therefore describe the context in some detail so that readers can judge to what extent their own context is similar to or different from the study context.
- *Construction of new knowledge*: The purpose of cooperative inquiry is to construct new knowledge through cycles of action and reflection. One way of judging the quality of the research is in the practical usefulness of this new knowledge. It should be clear that this knowledge is derived from the actions of the group and has been implemented practically. The way in which the final consensus of learning is constructed will also reflect on the quality of the inquiry.

### Ethical considerations

All of the usual ethical considerations apply,^3^ but there are some specific issues that arise in cooperative inquiry.^17^ Although members of the CIG will have given their consent to participate, the activities of the group will inevitably impinge on other people in the practice environment. Consent must, therefore, also be obtained before group members make observations of others or examine documents that were produced for another purpose. Confidentiality of all involved should be maintained.

Often the group is facilitated by a researcher who is also intending to write up the work for a thesis and publication. This intention must be made clear to the group members at the beginning and ownership of the findings and authorship explicitly discussed. Because of its participatory nature, the ‘development of the work must remain visible and open to suggestions from others’.^17^

## Conclusion

This article has described the key characteristics and types of participatory action research. It has then described in detail the methods of the cooperative inquiry group within the tradition of professional PAR. It is hoped that this will enable researchers to develop better research proposals using PAR as a study design.
